# Fast and Precise Non-Contact Measurement of Cylindrical Surfaces with Air Gauges

**DOI:** 10.3390/ma14133728

**Published:** 2021-07-02

**Authors:** Czeslaw Janusz Jermak, Michal Jakubowicz, Michal Wieczorowski, Miroslaw Rucki

**Affiliations:** 1 PROTiM SP. Z O.O., Trzebinska Street 6, 60003 Poznan, Poland; c.jermak@protim.pl; 2 Division of Metrology and Measurement Systems, Poznan University of Technology, Piotrowo Street 3, 60965 Poznan, Poland; michal.wieczorowski@put.poznan.pl; 3 Faculty of Mechanical Engineering, Kazimierz Pulaski University of Technology and Humanities in Radom, Stasieckiego Street 54, 26600 Radom, Poland; m.rucki@uthrad.pl

**Keywords:** cylindrical surface, air gauge, roundness measurement, accuracy

## Abstract

In this paper, the results of an investigation on the application of air gauges in the measurement of out-of-roundness parameters are presented. The principle of the measuring system is explained, in particular the novel design of the floating gauge head. An algorithm for fully automated measurement and data processing is described. The results from a series of initial measurements provided the data for further simulations, which revealed possible sources of errors. The simulations helped to evaluate the influence of some of the parameters on the final measurement results. After various accuracy tests, the method accuracy (MA) parameter was calculated in relation to the dedicated reference form tester. The result MA = 9.29% was judged to be highly satisfactory considering the short measurement time and non-contact method.

## 1. Introduction

In many industrial tasks, the measurement and assessment of cylindrical surfaces is necessary to obtain good performance for the assembled units [1]. The influence of the surface texture of honed cylinder liners on the tribological characteristics and performance of the piston ring–liner couple has been described in previous research [2]. Roundness affects the friction torque in the needle roller bearings [3]. Roundness and waviness deviations alter the vibrations generated by the rolling ball bearings [4]. The harmonic components of the roundness profile are very common in industry, where they are used for the description of the rotor’s geometry, since its variations cause local stiffness deviations [5]. The various frequency characteristic components result in different impacts on the operation of the ultra-precise shafts due to the manufacturing errors on the components’ surfaces [6]. As well as measurement and analysis of the surface topography of an engine cylinder—as a fingerprint of the manufacturing process [7]—it is also necessary to assess its meso- and macro-features: roundness and cylindricity.

Since the development of CMMs, evaluation of cylindrical and conical form errors has become a significant issue in precision coordinate metrology [8]. After a number of measurement points have been collected, the distances from the model, which may generate the form error, are calculated [9]. Even partial cylindrical surfaces may be examined using this method [10]. More accurate assessment requires significantly more time and resources, which in turn increases the overall cost of the operation.

Ever since the 1950s, air gauges have been considered as low-cost and simple devices for high-accuracy dimensional measurements. They are able to inspect single dimensions and perform differential measurements, which make them useful in automation [11]. Air gauging has all the advantages of non-contact measurement [12]. Over 60 years ago, Tanner [13] described the simplified measurements of lobing, circularity, and bore alignment with air gauges. Recent studies indicate that air gauging offers sufficient multiplication and reliability for tolerance measurement, significantly better than those of mechanical gauges [14], and it is still in use for surface topography characterization [15], form error measurement [16], and cylindrical surfaces assessment [17].

This study aimed to apply the advantages of air-fed, non-contact measurement in the accurate assessment of roundness and cylindricity. Its most valuable outcomes were two items: the automatic device “Geoform”, equipped with an innovative gauge head [18]. Thorough accuracy analysis consisted of simulations aimed at identification of error sources, then the capability of out-of-roundness measurement was checked, a similar check was made for cylindricity, and, finally, the overall method accuracy was determined.

This topic is an important one from the point of view of the study of surfaces, both at the macro and meso scales. A cylindrical surface was used as an example here, as it appears in many studies and has many applications. For the proper functioning of mechanisms and systems, it is very important to provide features in all scales, as these kinds of surfaces are often an effect of two processes and have to possess good properties under contact [19]. Pneumatic devices provide a very fast and accurate method, with the additional option of cleaning surfaces using a flow of air.

## 2. Materials and Methods

**Figure 1:**
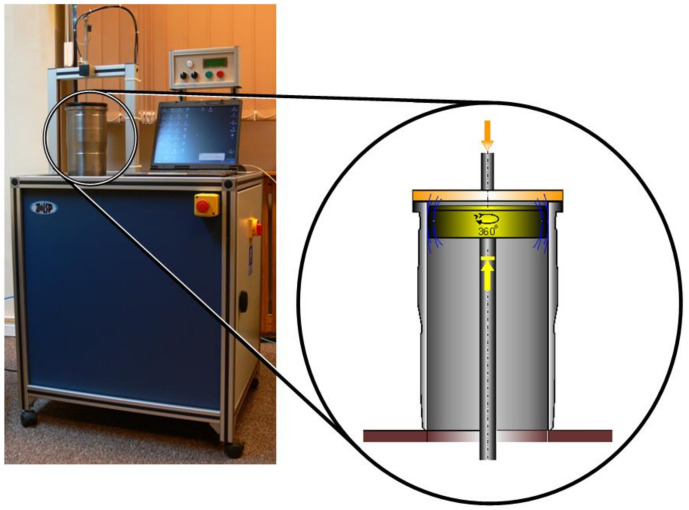
The Geoform measurement system with the measured cylinder.

The Geoform measurement system shown in Figure 1 was designed to perform measurements of diameters and the out-of-roundness of inner surfaces. During the measurement, the measuring head moves upwards to reach a certain height and then rotates in the range of 370°, simultaneously transmitting data on 4096 probing points. The operator can change the number of the measurement levels. As is usual procedure for air gauging, calibration with a setting ring should be undertaken before the measurement. After this procedure is complete, the results are presented in numerical form and as a circle diagram. The software also makes it possible to obtain the harmonics of the amplitudes at the actually measured level [20].

The Geoform measurement system is based on a “three-point” method described in several publications concerning the measurement of roundness [21,22,23,24]. The gauging head is equipped with three independent small-chamber air gauges. Two of them compile the reference points, like in typical three-point measurement, while the third one collects the measurement data of the profile. The values obtained from those three points are properly recalculated [18].

The main measurement task of the Geoform was roundness assessment at three positions inside the hollow cylinder. Its tolerances were defined, following [25,26], as *T_D_* = 10 µm in the middle and 15 μm in the upper and lower cross-sections. The gauge head was hidden inside the table and moved up inside the cylinder. It was not necessary to precisely adjust the cylinder’s position, because the gauging head is flexible and can self-center when entering the cylinder.

**Figure 2:**
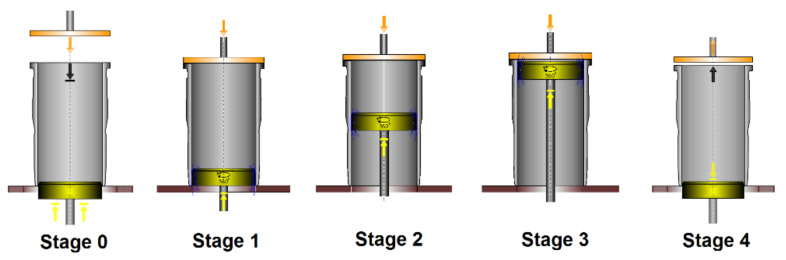
Stages of the measurement process.

The measurement is performed at three levels (bottom, middle, and top), with the gauge head performing a full revolution at each position. The procedure can be described in the following five stages, as shown in Figure 2.

- Stage 0:
  1. Initial position of the measuring head; the top edge is ca. 5 mm over the table surface.
  2. Manual positioning of the measured part over the gauge head edge.
  3. Automatic fixture of the part with the top cover.
- Stage 1:
  1. The measuring head moves up and stops at the first (lower) measurement position.
  2. The gauge head performs a full revolution while collecting measurement data.
  3. Data transmission to a PC.
- Stage 2:
  1. The gauge head moves to the position of the middle intersection.
  2. Revolution and measurement.
  3. Data transmission to a PC.
- Stage 3:
  1. The gauge head move to the upper position.
  2. Revolution and measurement.
  3. Data transmission to a PC.
- Stage 4:
  1. Retraction of the gauging head to the initial position.
  2. Release of the measured part.
  3. Data processing.

The actuator control system is connected to the microprocessor and microcontroller, which enable the communication between the pressure transducers of the air gauges, the control panel, and the main computer where the data is processed. The microcontroller’s software was designed using the Bascom language, while the control software for the PC was designed in the Basic language in the Windows environment, using the Visual Basic tool.NET (VB .NET).

**Figure 3:**
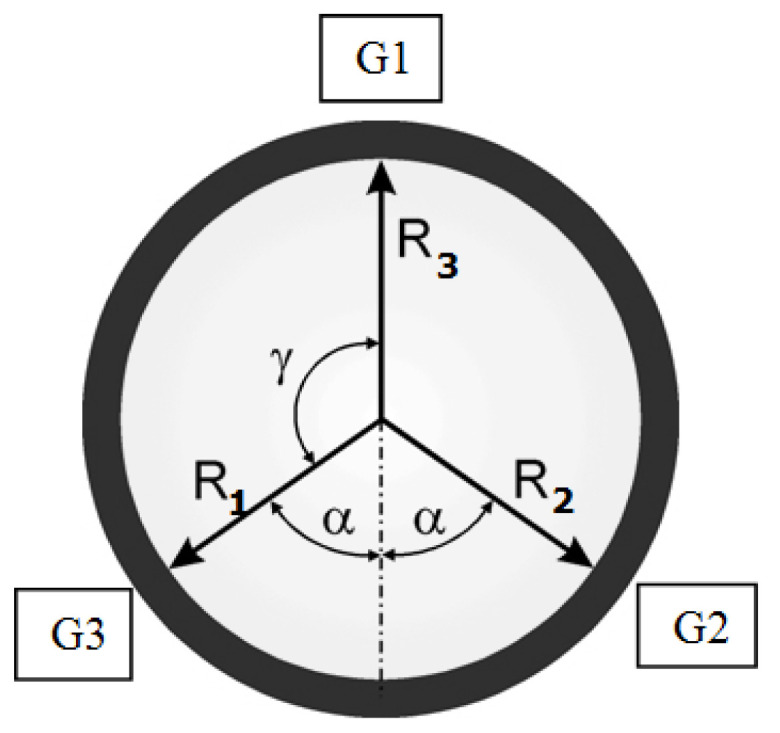
Positions of three separate air gauges, G1, G2, and G3, in the gauging head.

The novelty of the method employed in the Geoform measurement lies in the following:

- The gauge head is mounted on the flexible rod (a floating head).
- The gauge head contains three independent small-chamber air gauges, G1, G2, and G3, as shown in Figure 3. This is the principal difference compared to conventional solutions with three nozzles connected into one measuring chamber. Initial research proved that this solution offers certain advantages [27].
- In order to assess roundness, a novel algorithm designed for analysis of three independent signals is used.

Another novelty of the presented method is the concept of the floating gauging head. It is based on the three-point measurement discussed elsewhere [27].

## 3. Data Processing

Data processing can be divided into five main procedures: data collection, data filtration and smoothing, profile closing, interpolation, and calculation of the profile and roundness parameters.

### 3.1. Data Collection

Since the air gauges are the “eyes” that assess and indicate deviations in the measured feature [13], their static and dynamic characteristics require thorough analysis [28]. The following dimensions were used for the air gauge in the experiments. The inlet nozzle diameter was chosen to be *d_w_* = 1.020 mm, while the respective inner and outer diameters of the measuring nozzle were *d_p_* = 1.610 mm and *d_c_* = 4.8 mm, in order to ensure that the multiplication factor |*K*| = 0.5 kPa/μm with non-linearity below *δ* = 0.5%, in the measuring range *z_p_* = 100 μm. The chamber volume was set as *V_k_* ≈ 1.2 cm^3^, which provided good dynamic characteristics; the time constant *T* = 0.008 s; and the dynamic error below 5% at the input frequency of *f_0.95_* = 7 Hz. Importantly, because the inlet nozzles’ diameters were set, no regulation was needed prior to measurement, and the metrological characteristics remained unchanged. When the measurement task is different, the inlet nozzles can be replaced, providing more suitable characteristics for the application.

**Figure 4:**
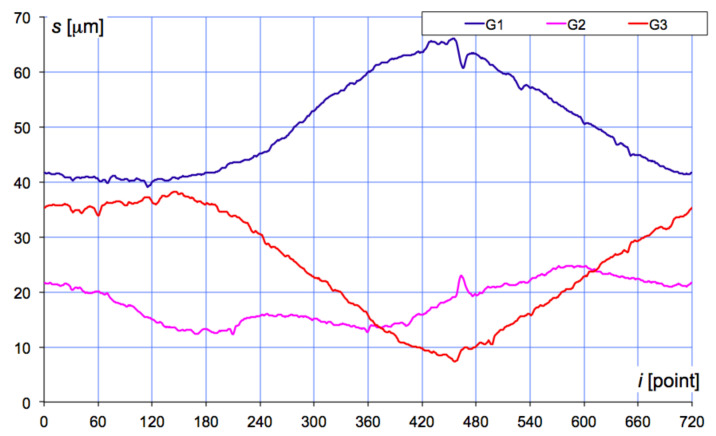
Displacement *s* registered by the air gauges G1, G2, and G3 during the 370° rotation of the gauging head.

Each of the air gauges G1, G2, and G3 placed in the gauging head was fed with stabilized and filtered pressured air of *p_z_* = 150 kPa. The back-pressure *p_k_* was measured with piezoresistive transducers (class 0.05) with an integrated AD converter and an independent memory buffer. Each gauge had its calibration data saved in the system’s memory. During the measurement, the gauge head performed a 370° rotation and *N* = 1000 measurement points were collected. The registered back-pressure *p_k_* (kPa) was then converted to displacement *s* (μm) values, in accordance with the air gauging principle. Figure 4 presents the displacement plots derived from the three air gauges at each point *i* during the rotation of the gauging head.

### 3.2. Data Smoothing, Profile Closure, and Interpolation

Filtration procedures must be chosen very carefully, since their improper use may be the cause of measurement errors [29]. Thus, thorough analysis of the filtration and smoothing procedures can help to improve the measurement results [30]. In the existing Geoform device, in order to eliminate the excess errors, the collected data were smoothed out, averaging the value of each point using the three preceding and three following points. Effectively, seven points were considered, as follows:

(1) $$y_{i}^{\prime }=\frac{1}{21}(-2y_{i-3}+3y_{i-2}+6y_{i-1}+7y_{i}+6y_{i+1}+3y_{i+2}-2y_{i+3})$$

where *y_i_* and *y*′*_i_* are the rough and smoothed coordinates, respectively.

This smoothing procedure was applied to eliminate the random errors. Moreover, it prepared the collected data for the subsequent numerical operations, such as interpolation and integration or differentiation. In the applied method, the least squares method was utilized, with the polynomial of the *j*-th degree fitted to the 2*n* + 1 values of the variable so that the sum of the squares of the distances between the calculated *y*(*x_i_*) and the initial *y_i_* values was minimal. It should be noted, however, that the higher the polynomial degree is, the larger the number of points that must be taken into consideration, and the respective curve is less smooth. The same procedure can be repeated for a particular dataset and the number of repetitions chosen empirically.

Next, the number of the points in the analyzed profile was reduced down to 1024 through the calculation of the ordinate between the existing points for the specified abscissa. Bessel’s interpolation formula offered a reduced computation cost and simplicity at an acceptably small error of interpolation.

Keeping in mind that the specific task of the data processing aimed at out-of-roundness assessment, 50 cylinders were measured with a Taylor Hobson Talyrond 365 reference device, each in two intersections. A filtration algorithm was chosen to satisfy the requirements of the measurement. Moreover, the measuring head geometry was modified accordingly after the dominating harmonics of these specific parts were derived from the measurements. In addition, it was found that some typical dirt particles generated peaks in the measured profiles and introduced some distortions into the obtained results. In order to eliminate this effect, filtration was applied to remove harmonics #0 (average) and #1 (eccentricity) from the full profile, and only then was the out-of-roundness parameter calculated.

Equation (1) is based on the approximation of the second degree polynomial, which provided fairly good results. Preliminary research revealed that it was not necessary to apply more accurate, but far more complicated, functions. All obtained points from *y_4_* to *y*_*N*−3_ were subjected to this procedure.

As the gauge head is non-stationary (it has no fixed axis), it was possible that the start and finish points after the 360° revolution might not have the same coordinates. To address this issue, the algorithm needed to be modified in order to receive the closed profile where the first and the last points had the same coordinates.

The “profile closure” procedure was performed after the measurement of 370° was completed. Then, the difference between the coordinates of the points *y_365°_* and *y_5°_* was divided by the number of points in between. The coordinates of each of the points were then corrected with the calculated value, so that the first and last corrected points coincided.

The main purpose of the interpolation applied here was to obtain the reduced number of 720 points for analysis, as shown in Figure 4 above. The formula was based on Bessel’s theoretical assumptions [31]:

(2) $$f(x)=f(x_{0})+k\Delta_{0}-k_{1}(\Delta_{1}-\Delta_{-1})$$

where $\Delta_{-1}=y_{0}-y_{-1}$; $\Delta_{0}=y_{1}-y_{0}$; $\Delta_{1}=y_{2}-y_{1}$; $k=\frac{x-x_{0}}{h}$ $k_{1}=\frac{k(1-k)}{4}$; $h=x_{0}-x_{-1}=x_{1}-x_{0}=x_{2}-x_{1}$.

### 3.3. Calculation of the Profile and Roundness

**Figure 5:**
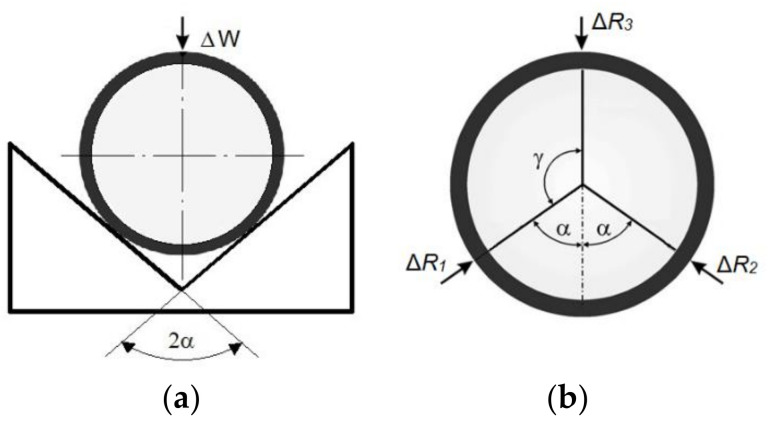
Three-point roundness assessment: (**a**) with the V-block method; (**b**) with the three-point floating gauge head.

It has been demonstrated elsewhere that the V-block method is highly suitable for accurate cylindricity measurements [24]. In the case of a floating gauge head, additional calculations should be performed to achieve results comparable to the typical V-block measurement. Figure 5 compares V-block roundness assessment, obtaining the Δ*W* value, with the corresponding measurement using three gauges, obtaining Δ*R*_1_, Δ*R*_2_ and Δ*R*_3_ indications.

In the Geoform gauging head, the two fixed points usually used in the V-block method were replaced with two air gauges (G2 and G3, as shown in the Figure 4) that also provided measurement data. To obtain the virtual value Δ*W* (of the V-block measurement) from the points collected with the air gauges G1, G2, and G3, the following formula was derived from [32]:

(3) $$\Delta W=\Delta R_{3}+\frac{\Delta R_{1}+\Delta R_{2}}{2\cos\alpha }$$

where Δ*R_1_*, Δ*R_2_*, and Δ*R_3_* denote changes of indications from G3, G1, and G2, respectively, and 2α indicates the angle shown in Figure 5b between the measurement radiuses *R_1_* and *R_2_*.

**Figure 6:**
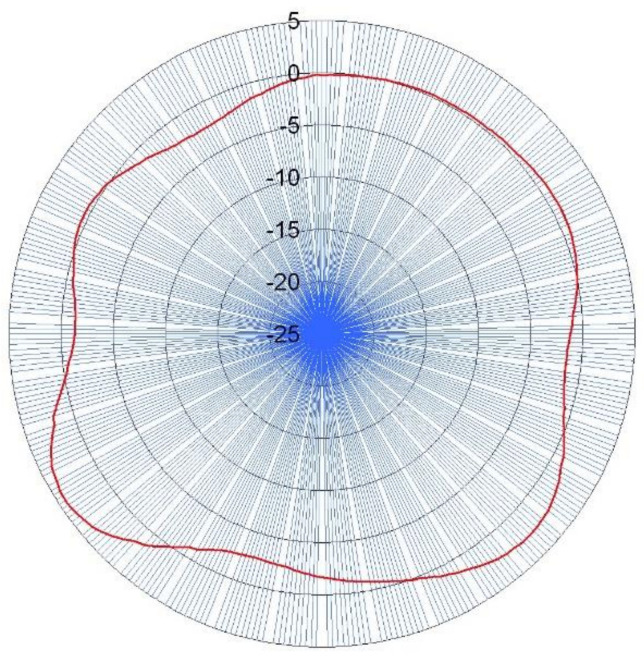
Roundness profile derived from variations of indications from the gauges G3, G1, and G2 respectively.

Then, Fourier analysis had to be applied in order to obtain the roundness profile, and the particular harmonics underwent a correction procedure. The Fourier coefficient calculation was derived from the trapezoid function replacement in Euler’s formulas [33]. Figure 6 shows an example of the profile obtained from the three signals G1, G2, and G3. 

For the approximate analysis considering the 15th harmonic, the following equations were attained [34]:

(4) $$na_{m}=\sum_{k=0}^{2n-1}\Delta W_{k}\cos\frac{km\pi }{n}$$

(5) $$nb_{m}=\sum_{k=0}^{2n-1}\Delta W_{k}\sin\frac{km\pi }{n}$$

where *m* = 2, 3, …, 15 and *k* = 0, 1, 2, …, 2*n* (in this case, *n* = 360, since the rotation angle was 360°).

Then, the particular harmonics’ amplitudes could be calculated as follows:

(6) $$A_{kp}=\sqrt{a_{m}^{2}+b_{m}^{2}}$$

Variations of the impact of the harmonic amplitudes on the value Δ*W* can be expressed by the harmonic suppression correction *U_k_*, as described by the following equation [34]:

(7) $$U_{k}=1+\frac{\cos k(180-\alpha )}{\cos\alpha }$$

In practice, however, it is most likely to amplify measurement noise. To solve this problem, the initial measurement of a typical component should be done to detect the expected harmonics characteristics. The true amplitude can then be calculated from the next equation:

(8) $$A_{kr}=\frac{A_{kp}}{U_{k}}$$

The actual differences between the radius and its measurement results can be calculated from the Fourier series synthesis, as follows:

(9) $$\Delta R_{k}=\sum_{m=2}^{15}A_{krm}\sin(km+\psi_{m})$$

where *m* = 2, 3, …, 15 and *k* = 0, 1, 2, …, 2*n* (in this case, *n* = 360, since the rotation angle was 360°).

When the point-by-point calculation is finished using Equation (9), the actual out-of-roundness *RON* can be described as the difference between the maximum and minimum obtained radiuses:

(10) $$RON=\Delta R_{\max}-\Delta R_{\min}$$

## 4. Simulations and Measurement Results

The specialized software was developed to determine the coefficients of identification, and to reconstruct the actual profile from the measurement data.

In order to evaluate the conformity of the obtained results, a series of simulations and reference measurements were carried out.

**Figure 7:**
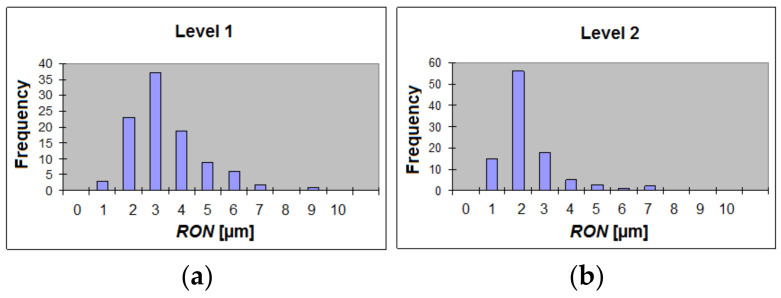
Histograms of out-of-roundness *RONt* achieved for: (**a**) Level 1; (**b**) Level 2.

The initial model prepared for the tests of the proposed algorithms was based on the preliminary laboratory measurement of 100 cylinders manufactured for the automotive industry using the Talyrond 365 reference device. Since the roundness tolerance of the cylinders was set as *T_1_* = 15 µm close to edges and *T_2_* = 10 µm in the middle cross-section, all cylinders were measured at these three levels, denoted S1, S2, and S3 in the Table 1. The actual measured values of the roundness deviation appeared to be much smaller than the roundness tolerance *T_D_*: they were no larger than 9.5 µm for the top and bottom positions (levels 1 and 3) and did not exceed 7.0 µm in the middle (level 2). The distribution was asymmetric, with skewness varying between 1.4 and 2.6. In Table 1, the statistical parameters of the analyzed population are presented and examples of histograms for different levels are shown in Figure 7.

**Figure 8:**
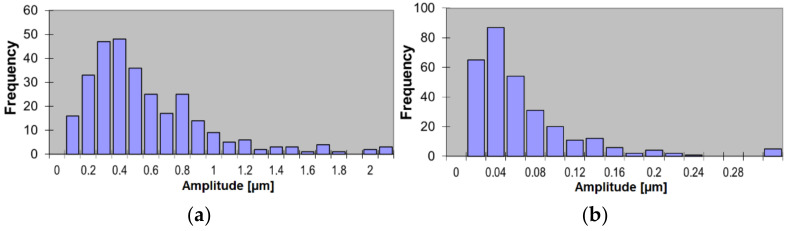
Histograms of roundness deviation *RONt* measured on 100 cylinders: (**a**) second harmonic; (**b**) fifth harmonic.

With regard to Figure 7, it should be noted that only the overall spectrum character was similar at different levels. The dominant harmonic shifted from *RON* = 3 μm at level 1 to *RON* = 2 μm at level 2. Moreover, the maximum frequency at level 2 was ca. 35% higher than at level 1. Examples of histograms obtained for the second and fifth harmonics are shown in Figure 8.

The highest amplitudes were achieved for the second harmonic, at 2.6 µm, and for the third, at 0.98 µm. The distribution types of the amplitudes of the fourth, fifth, and seventh harmonics were found to be similar, and their maximal value was 0.56 µm, while the mean values were below 0.08 µm. Naturally, when the frequency array is expected to change (new series of details, new tool, new technology etc.), the preliminary identification of the expected harmonics should be performed.

**Figure 9:**
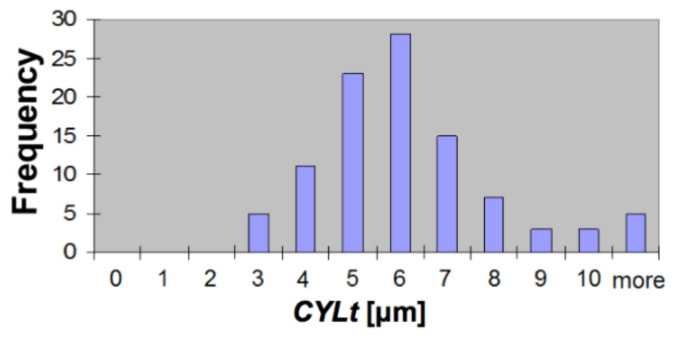
Histogram of cylindricity deviation *CYLt*.

Additionally, the cylindricity deviation was calculated based on the data from Talyrond 365 measurements. The cylindricity deviation value *CYLt* varied from 2.14 µm to 10.4 µm with small asymmetry (skewness = 0.81). The results of the measurements are collected in Table 2 and Figure 9 presents the histogram of the cylindricity deviation parameter *CYLt*.

Since the floating gauge of the Geoform device head was not able to repeatedly align itself with the cylinder’s axis at the measurement positions, the mean square circles could not refer to said axis. Therefore, the cylindricity was assessed in a simplified way, based on the maximum and minimum roundness deviation values for the measured cylinder, regardless of the current measurement level. Since this method did not take the position of the actual axis into account, a reference measurement of the cylindricity had to be performed as well. This revealed that the cylindricity deviation values derived with the simplified method *CYLt_s_* were smaller by 0.12 ÷ 1.11 µm than the results from the reference measurements *CYLt_r_*. The mean value of difference *CYLt_r_*–*CYLt_s_* was 0.69 µm and the standard deviation was 0.39 µm.

The measurement results were subjected to a data evaluation process using the algorithm described in the Section 3 in order to choose appropriate factors and coefficients. During the simulations, two types of random error were generated and added to the input data [35].

The first type of error was the direct air gauge measurement error *e_ag_*. Its expected value was E[*e_ag_*] = 0 with standard deviation *s* = 0.5 µm. A total of 1000 measurements were simulated in order to assess the impact of this error type on the obtained roundness deviation values *RONt* and the amplitudes of the particular harmonics.

The second type was the displacement of the gauge head axis in relation to the cylinder’s axis. Despite the self-centering characteristic, common among three-point air gauging measuring heads, such a displacement can still occur. The slot between the gauge head surface and the inner surface of the measured cylinder suggested a possible displacement of ±8 μm. Moreover, it was assumed that during the gauge head revolution a random value of the axis displacement between the two subsequent measurement points would follow the normal distribution, with a standard deviation of 0.1 µm radially and 2° angularly.

**Figure 10:**
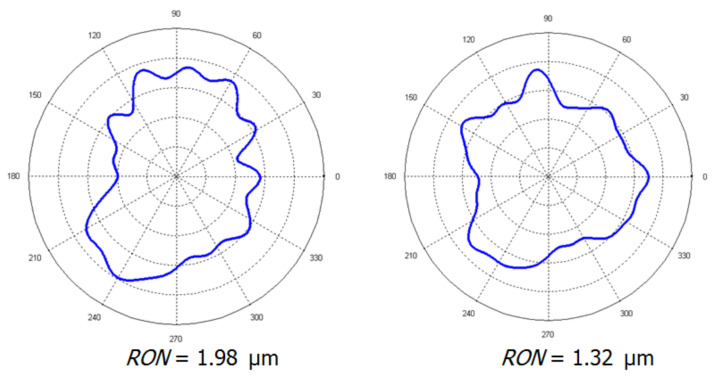
The profiles used for the air gauge positioning precision simulation.

Simulations also made it possible to investigate the impact of the air gauges position deviation in the measuring head. It was assumed that the deviation of the angles *α* and *γ* between the air gauges (see Figure 3) could be kept within the range of ±0.5°. All possible scenarios for each air gauge position error in the measuring head were subjected to the test based on two reference profiles with roundness deviations of 1.98 µm and 1.32 µm, as shown in Figure 10.

The simulations revealed that the predicted occurrence of random errors had a negligible effect on the final measurement results. The maximum difference between the input (true value of *RONt*) and output (received after data processing according to the tested algorithm) values never exceeded 0.20 µm. The mean value of *RONt* differed from the known true value by between 0.026 µm and 0.092 µm, which was well below the resolution of the used air gauges. The standard deviation of the errors’ values varied from 0.076 µm to 0.100 µm. When the axis displacement error was added to the array of random errors, the results changed by less than 0.05 µm. The histograms of the deviations showed no asymmetry; however, in several of iterations of the simulation, the random error introduced significant deviation in the harmonic’s amplitude.

**Figure 11:**
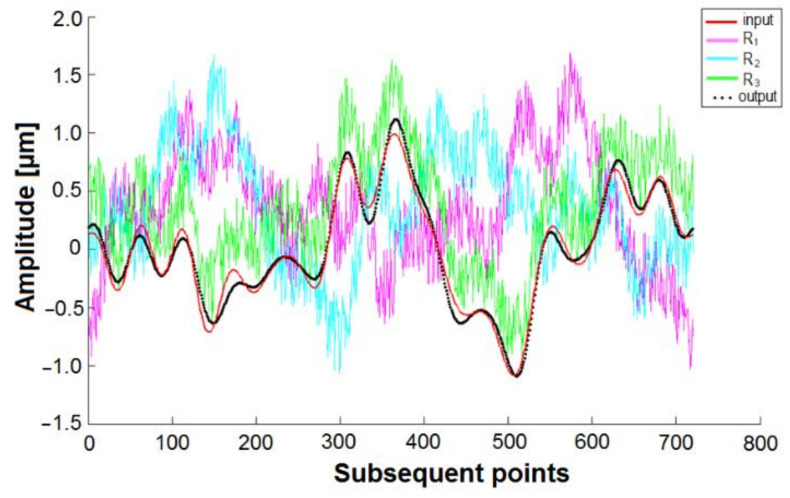
Example of the random error simulations.

An example of the simulation results is shown in Figure 11, where “input” corresponds with the true values of the simulated profile, *R_1_*, *R_2_*, and *R_3_* represent the measurement signals received from the air gauges G1, G2, and G3, respectively (random errors were taken into account), and, lastly, “output” represents the processed data obtained from the simulated measurement.

While the measurement system revealed good insensitivity to the deviations of the *γ* angle, the angle *α*, both by itself or combined with *γ*, had a larger impact on the measurement results. The final results, however, differed from these situations and had no angle errors less than 0.1 µm, which is negligible anyway.

## 5. Accuracy Analysis

As roundness is a complicated characteristic affected by a multitude of factors, statistical estimation of uncertainty is problematic. To address this issue, the proposed roundness measurement system Geoform was subjected to accuracy evaluation based on the method proposed by Adamczak et al. [36], with the results from the tested system compared to the corresponding results obtained a the method of higher accuracy, i.e., the reference method. The reference data were obtained using a Taylor-Hobson Talyrond 365, which is capable of data sampling of up to 200,000 points with 0.25 µm resolution. Thus, the relative error of the roundness deviation assessment *w_RON_* can be calculated as follows:

(11) $$w_{RON}=\frac{RONt_{m_{i}}-RONt_{a_{i}}}{RONt_{a_{i}}}$$

where $RONt_{m_{i}}$ is the roundness deviation value from the tested method in the *i*-th point and $RONt_{a_{i}}$ is the corresponding value calculated from the reference method.

The relative error *w_RON_* covers all the component errors that occur in the measurement system, both random and systematic. Among the main sources of detectable errors, the following should be pointed out in particular:

(a) Measurement signals generated by air gauges (non-linearity of characteristics, air flow instability, etc. [37]), pressure transducers, AD converters, and the geometrical inaccuracy of the gauge head and other components.
(b) The air feed and, above all, pressure instability.
(c) Environmental sources, temperature in particular.
(d) The gauge head angle indication.
(e) The data processing procedures, such as simplified formulas, rounding the values, etc.

In the statistical analysis, estimation and significance tests were performed for the mean values, variance, and standard deviation. The confidence intervals for the mean value were determined as follows:

(12) $$\left({\bar{w}}_{RON}-u_{p}\frac{s}{\sqrt{n}};{\bar{w}}_{RON}+u_{p}\frac{s}{\sqrt{n}}\right)$$

where *s* is the standard deviation and *u_p_* is the quantile of the standardized normal deviation; for P = 0.95, it is *u_p_* = 1.96.

The final accuracy of the method was assessed for the confidence level most common in industrial measurements [38]. From the mean value of all measured points ${\bar{w}}_{RON}$, the method accuracy *MA* [36] can be calculated:

(13) $$MA=\left|{\bar{w}}_{RON}\pm u_{p}s\right|\cdot 100\%$$

The significance test for the mean value was performed for *n* measurements at the confidence level of *α* = 0.05. The zero hypothesis was set as follows:

(14) $$H_{0}:{\bar{\Delta }}_{MP(e)}={\bar{\Delta }}_{0}$$

where ${\bar{\Delta }}_{0}$ is the error of the reference method, assumed to be ${\bar{\Delta }}_{0}$ = 0.02.

The alternative hypotheses were set as follows:

(15) $$H_{0}:{\bar{\Delta }}_{MP(e)}\neq \;{\bar{\Delta }}_{0}\;\mathrm{or}\;H_{2}:{\bar{\Delta }}_{MP(e)}<{\bar{\Delta }}_{0}\;\mathrm{or}\;H_{2}:{\bar{\Delta }}_{MP(e)}>{\bar{\Delta }}_{0}$$

The results of the statistical analysis are presented in the Table 3.

Since the accuracy of a measurement device used for measurement of a geometrical surface structure should lie within the range of 10–25% [36], the obtained value of 9.29% appears to be a very good result. With this level of accuracy, along with its relatively low price and swift operation, the Geoform device should prove to be a very competitive measurement system for roundness assessment.

## 6. Conclusions

In this study, air gauges were applied in the fully automatic measurement system Geoform, which was designed for the assessment of the inner surfaces of cylinders manufactured for internal combustion engines. One of the important findings was the measurement capability of the floating three-point gauge head combined with an algorithm, which could recalculate the measurement results to values similar to those of the V-block method in order to obtain the roundness deviation values.

The measurement system and its subsystems were subjected to thorough metrological analysis. The simulations were carried out to highlight the errors generated by the data processing procedures and the most important geometrical factors. The simulated impact on the measurement results was found to be negligible.

The overall accuracy of the proposed method was investigated through comparative analysis, which demonstrated that the method accuracy *MA* = 9.29%. This was judged to be highly satisfactory, since the accuracy of a measurement device used for measurement of a geometrical surface structure should lie within the range of 10–25%. The Geoform system proved to be capable of performing accurate, cost-effective, and relatively fast non-contact roundness measurements. In the near future, thorough analysis of the filtration and smoothing procedures is planned to further improve the measurement results. 

## Figures and Tables

**Table 1:** Results of Talyrond measurement of the cylinders [34].

Out-of-Roundness *RON*	S1 (μm)	S2 (μm)	S3 (μm)
Mean $\bar{RONt}$	2.93	1.88	2.08
Standard deviation *s*	1.40	1.17	1.33
Skewness *Skew[RONt]*	1.31	2.43	2.56
Minimum *RONt_min_*	0.79	0.54	0.65
Maximum *RONt_max_*	8.51	6.94	9.45

**Table 2:** Statistical data of the cylindricity deviation value measured with the Talyrond 365.

Out-of-Cylindricity *CYLt*	(μm)
Mean $\bar{CYLt}$	5.58
Median *m*	5.35
Standard deviation *s*	1.90
Skewness	0.81
Minimum	2.14
Maximum	10.40

**Table 3:** Statistical analysis of the measurement system.

Population	100	Standard Deviation	0.00008
Maximum and minimum values of the relative error *w_RON_*	0.092/0.085	Confidence interval of an error of the method	0.088 ± 0.022
Mean value ${\bar{w}}_{RON}$	0.088	Variance for the sample	0.0000064
Confidence interval of the mean value	0.0880 ± 0.0022	Overall accuracy *MA*	9.29%

## Data Availability

Data available on request due to privacy restrictions.
